# Folic Acid Reduces Insulin Resistance in Mice With Diet‐Induced Obesity by Altering One‐Carbon Metabolism and DNA Methylation Patterns of Hypothalamic and Hepatic Insulin Receptor Gene

**DOI:** 10.1002/mnfr.70181

**Published:** 2025-07-16

**Authors:** Eva Kranenburg, Ruslan Kubant, Clara E. Cho, Zeyu Yang, Mahaylia Datars, Jianzhang Dong, G. Harvey Anderson

**Affiliations:** ^1^ Department of Nutritional Sciences Temerty Faculty of Medicine University of Toronto Toronto Ontario Canada; ^2^ Department of Human Health Sciences College of Biological Science University of Guelph Guelph Ontario Canada; ^3^ Department of Physiology Temerty Faculty of Medicine University of Toronto Toronto Ontario Canada

**Keywords:** DNA methylation, folic acid, insulin resistance, obesity, one‐carbon metabolism

## Abstract

Diet‐induced obesity and insulin resistance (IR) are associated with alterations in one‐carbon (1C) metabolism, gene methylation, and expression. Folate, a methyl donor in 1C metabolism, is essential in gene methylation and expression and has been shown to reduce IR but the precise mechanism(s) remains unclear. Therefore, we investigated whether reduced IR can be explained by 1C metabolism modifications and differences in hypothalamic and hepatic IR‐related genes expression and methylation patterns. Four‐week‐old male C57BL/6J mice (*n* = 12/group) received high‐fat diets (HFDs) with 1‐ (control), 5‐ (5FA‐HFD), or 10‐fold (10FA‐HFD) AIN‐93G amounts folic acid (FA) for 15 weeks. Body weight, hepatic 1C metabolites, plasma insulin and glucose, and hepatic and hypothalamic expression and methylation of IR‐related genes were measured. 5FA‐HFD and 10FA‐HFD mice had ∼50% lower HOMA‐IR compared to control. 10FA‐HFD mice also had lower body weight (9%) and adiposity (13%) and higher s‐adenosylmethionine levels (19%). 5FA‐HFD mice had higher hepatic s‐adenosylhomocysteine levels (26%) and DNA methyltransferase 3b expression (45%). Methylation of the *InsR* gene was correlated with gene expression and the improved metabolic phenotype. FA supplementation reduced IR by modifying 1C metabolism in DIO male mice by differentially altering hypothalamic and hepatic methylation patterns of *InsR* gene.

## Graphical abstract

Folic acid (FA), a methyl donor in one‐carbon (1C) metabolism, is essential for gene methylation and expression and reported to reduce insulin resistance (IR). However, whether reduced IR is mediated by insulin receptor (*InsR*) gene methylation is unknown. FA supplemented to high‐fat diet fed mice reduced body weight, adiposity, IR, and altered 1C metabolism. This was associated with hepatic and hypothalamic *InsR* methylation and expression. This highlights the potential to leverage 1C metabolism to reduce obesity progression by gene methylation.

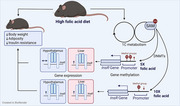

**Abbreviations**
1C: one‐carbon
CpG: cytosine‐phosphate‐guanine
DIO: diet‐induced obesity
DNMTs: DNA methyltransferases
FA: folic acid
HFD: high‐fat diet
HOMA‐IR: homeostatic model of assessment for insulin resistance
IR: insulin resistance
SAH: s‐adenosylhomocysteine
SAM: s‐adenosylmethionine

## 1 Introduction

The obesity epidemic remains a global crisis with adult overweight and obesity rates expected to reach 54% by 2035 [1]. Obesity is often associated with systemic dysregulation of metabolism, including insulin resistance (IR) and impairment of one‐carbon (1C) metabolism [2]. Its development is linked to numerous genes [3], with their expression and function impacted by changes in the epigenome [4, 5].

The epigenome refers to chemical modifications made to DNA and histone proteins which determine patterns of gene expression in the absence of changes to the DNA sequence [6]. These chemical modifications include DNA and histone methylation, acetylation, and non‐coding RNAs. DNA methylation involves the transfer of a methyl group donated from universal methyl donor, *S*‐adenosylmethionine (SAM), onto cytosine residues within cytosine‐phosphate‐guanine (CpG) dinucleotides by DNA methyltransferases (DNMTs). Methylation patterns are modifiable by environmental factors, including nutrients that modify 1C metabolism.

Folic acid, used in animal diets, is a synthetic form of folate and requires conversion to its active form, methyltetrahydrofolate (5‐MTHF), to be biologically functional. This conversion is crucial for various metabolic processes including the production of SAM, a key molecule involved in methylation reactions. FA has been shown to modify 1C metabolism and global DNA methylation patterns in both rodents and humans [7, 8]. In rodents, high (3.5 mg/kg diet, 1.75‐fold AIN‐93) to very high (40 mg/kg diet, 20‐fold AIN‐93) doses of FA were associated with increased global DNA methylation levels [8]. In humans, supplementation of 400 µg (1‐fold RDA) to 5 mg (12.5‐fold RDA) shows some evidence of altered global DNA methylation status, although these findings are heterogenous and inconclusive [8]. However, global methylation changes are considered less functionally relevant than gene‐specific methylation changes, which directly influence the expression of individual genes [9].

Modification of the FA content in gestational and postweaning rodent diets has been shown to affect various metabolic parameters, including body weight, glucose homeostasis, and IR [10, 11, 12, 13]. Impaired glucose homeostasis and IR have been linked to reduced proximal insulin signaling, including insulin receptor (*InsR*) gene expression as well as downstream mediators, such as phosphoinositide 3‐kinase (*Pi3k*) and *Akt* [14]. These effects occur across different tissues, including the brain and liver. The brain, particularly the hypothalamus, is an insulin sensitive organ which exerts systemic effects by regulating glucose homeostasis and food intake [15]. For instance, downregulation of hypothalamic *InsR* gene induces hyperphagia and IR [16]. Moreover, gestational high‐fat feeding alters methylation patterns of hypothalamic *InsR* with decreased gene expression levels in male offspring [17, 18]. We have previously shown altering the FA content of gestational diets impacts gene expression and methylation of hypothalamic energy regulatory genes in male offspring, including *InsR* [10, 11]. Similarly, the liver is essential in regulating whole body insulin sensitivity, as liver‐specific knockout of *InsR* abolishes insulin receptor substrate binding to PI3K, dysregulating insulin signaling and inducing IR [19]. In IR, the liver epigenome is dysregulated with differential methylation of genes in the insulin signaling pathway [20]. Altering FA content in the postweaning diet alters the expression and methylation of genes in the liver [13, 21, 22], although its effects on IR‐related genes are not known.

The purpose of this study was three‐fold: (a) to describe the effects of FA supplementation on 1C metabolism, IR, and changes in expression of IR‐related genes; (b) to examine if changes in gene expression in response to FA supplementation can be explained by differences in DNA methylation patterns of the genes; and (c) to determine whether methylation changes are tissue‐ and CpG site‐specific. We hypothesized that increasing the FA content would reduce HFD‐induced IR by altering 1C metabolism and DNA methylation patterns of IR‐related genes in the hypothalamus and liver.

## 2 Experimental Section

### 2.1 Experimental Design and Diets (Dosage Information)

**FIGURE 1:**
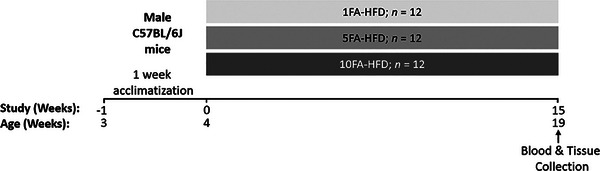
Schematic of the experimental design and timeline. All diets contained a 45 kcal% fat content with folic acid added in 1‐ (2.4 mg/kg, control), 5‐ (12 mg/kg), and 10‐fold (24 mg/kg) AIN‐93G recommended amounts. [23]

Experimental procedures were approved by the University of Toronto Institutional Animal Care and Use Committee (Protocol No. 20012871). Thirty‐six 3‐week‐old male C57BL/6J mice (Stock No: 000664) were purchased from Jackson Laboratory (Bar Harbor, ME, USA) and housed in ventilated plastic cages in groups of four on a 14:10‐h light‐dark cycle (lights on at 06:00 at 23°C ± 3°C) with food and water provided ad libitum. Following 1 week acclimatization on a high‐fat diet (HFD; 45 kcal% fat, Research Diets, Cat. No. D12451), mice were randomized to one of three dietary interventions (*n* = 12/group) for 15 weeks (Figure 1): (1) HFD with AIN‐93G recommended micronutrients with folic acid (FA) at 2.4 mg FA/kg diet (control; 1FA‐HFD), (2) HFD with 5‐fold FA (5FA‐HFD; 12 mg FA/kg diet), or (3) HFD with 10‐fold FA (10FA‐HFD; 24 mg FA/kg diet). Diets were kept in the dark at –20°C until being freshly provided to the animals on a weekly basis.

All three experimental diets were based on the American Institute of Nutrition‐93G (AIN‐93G) [23] diet formulation and were isocaloric with similar macronutrient, vitamin, and mineral amounts, differing only in the amounts of folic acid (Table S1). The amount of folic acid in the control diet was 2.1 mg per 4000 kcal or 2.4 mg/kg of diet. Folic acid was added in 5‐ (12 mg/kg) and 10‐folds (24 mg/kg) the control amount, in the 5FA‐HFD and 10FA‐HFD groups, respectively. This amount reflects the AIN‐93G recommended amounts (2.0 mg/kg diet) with adjustments made for energy density to contain a similar amount of FA per 100 kcal as the standard AIN‐93G diet. These doses have previously been shown to be non‐toxic and safe for rodents [10, 11, 22] with no adverse effects on the metabolic parameters examined, including food intake and body weight.

At 19 weeks of age (15 weeks on diets), mice were sacrificed following a 6‐h daytime fast (food removed between 07:00 and 09:00) via cardiac puncture while under isoflurane. Blood was collected and centrifuged to separate plasma, which was stored at –80°C. To assess the development of obesity, epididymal (as a proxy for visceral) and inguinal (as a proxy for subcutaneous) white adipose tissues were weighed and summed for total white adipose tissue mass. The whole brain was excised and frozen on dry ice. The liver was snap frozen in liquid nitrogen and stored at –80°C for further analyses.

To better understand how tissue‐ ‐and CpG‐site‐specific changes in gene expression and function impact the development of obesity and its comorbidities, we have utilized mouse high‐fat diet‐induced obesity (DIO) model which recapitulate many metabolic characteristics of human obesity, including IR [24] and impaired 1C metabolism [2]. We used male C57BL/6J mice as they are more susceptible to DIO than females to demonstrate proof of concept; however, we acknowledge that effects in females may differ and will be explored as a separate study.

### 2.2 Fasting Plasma Insulin and Glucose Measures

Plasma insulin concentrations were measured using a commercially available ELISA kit (Limit of detection = 0.06 ng/mL, Cat. no. 80‐INSMS‐E01, ALPCO, New Hampshire, USA). Plasma glucose concentrations were measured using an Analox GM9 Glucose Analyzer (Stokesley, UK). Fasting plasma insulin and glucose concentrations were utilized to calculate insulin resistance by proxy of HOMA‐IR [25], using the following formula:

$$\mathrm{HOMA}-\mathrm{IR}=\mathrm{fasting}\mathrm{glucose}\left(\mathrm{mmol}/L\right)*\mathrm{fasting}\mathrm{insulin}\left(\mathrm{mIU}/\mathrm{mL}\right)/22.5$$

### 2.3 One‐Carbon Product Concentrations

One‐carbon product concentrations were measured in the liver as it is a key site for *S*‐adenosylmethionine (SAM) formation and ∼85% of methylation reactions occur in this organ [26]. Concentrations of SAM, *S*‐adenosylhomocysteine (SAH), cystathionine, and methionine in the liver were analyzed by liquid chromatography with tandem mass spectrometry (LC‐MS/MS) at the Center of Metabolomics, Baylor Scott & White Research Institute, Dallas, TX, USA, as described elsewhere [27].

### 2.4 Relative mRNA Expression in Liver and Hypothalamus

The hypothalamus was excised from frozen whole brain as previously described [11]. RNA was extracted from the right side of the hypothalamus (∼4 mg of frozen tissue) by RNeasy Lipid Tissue Mini Kit (Qiagen; item no. 74804) according to the manufacturer's instructions. For RNA extraction in the liver, tissue was pulverized by metal tissue pulverizer (Cole‐Parmer; item no. RK‐36903‐05) in the presence of liquid nitrogen. Samples (∼30 mg frozen liver) were then homogenized with TRIzol reagent (Invitrogen, Grand Island, NY, USA) followed by chloroform extraction. A NanoDrop 2000 Spectrophotometer (Thermo Fisher Scientific, Inc., Waltham, MA, USA) was used to quantify 500–1000 ng of RNA (purity of RNA was evaluated by absorbance at the 260/230 and the 260/280 nm spectrum) for cDNA synthesis using the High‐Capacity cDNA Archive Kit (Applied Biosystems Inc.; ABI, Foster City, CA, USA) on the VeritiPro 96‐Well Thermal Cycler (Thermo Fisher Scientific, Inc., Waltham, MA, USA). An equal volume of cDNA (0.5 µL/sample) was then added to TaqMan Master Mix (Thermo Fisher Scientific, Inc., Waltham, MA, USA) with gene‐specific probes (Table S2) and ran on a QuantStudio 5 Real‐Time PCR System (Thermo Fisher Scientific, Inc., Waltham, MA, USA). Quantification was done using beta‐2 microglobulin (*B2m*) as an endogenous control for hypothalamus and TATA‐box binding protein (*Tbp*) for liver, which were selected based on preliminary screening for lowest variation. Results of genes of interest in targeted tissues were expressed as fold‐change by the 2^−△△CT^ (cycle threshold) method [28].

To assess the tissue‐specific effects of FA on insulin resistance, three key genes (*InsR*, Pi3k regulatory subunit 1, and *Akt1*) upstream in the PI3K/AKT signaling pathway were measured in the liver and hypothalamus. These genes were selected as they play a direct role in IR and encode for key components upstream in the signaling cascade [29]. Moreover, we have previously shown that changes in the gestational folate content of the diet leads to alterations in hypothalamic expression of genes in this pathway, most notably *InsR* [11]. Epigenetic regulation of hepatic insulin resistance has also been noted in response to diet‐induced obesity [20], and FA has been shown to alter methylation patterns of hepatic genes; therefore, the methylation patterns in this organ may be responsive to FA supplementation.

### 2.5 Gene‐Specific DNA Methylation

Genomic DNA was extracted from the liver (∼30 mg) and left side of the hypothalamus (∼4 mg) with the DNeasy Blood and Tissue Kit (Qiagen, Valencia, CA, USA; Item no. 69504), according to the manufacturer's instructions. DNA samples were quantified for normalization using an Epoch Spectrophotometer (Agilent BioTek, Santa Clara, USA) and 500 ng of DNA was bisulfite treated using the EZ DNA Methylation‐Lightning Kit (Zymo Research; item no. D5030).

Candidate gene(s) for methylation analysis were determined based on differential gene expression in the liver and hypothalamus in response to FA supplementation. The targeted CpG island was identified based on proximity to the first exon of the candidate gene using the University of Santa Cruz Genome Browser (GRSC m39/mm39). Five microliter of bisulfite‐treated templates were amplified using 0.02 U/µL Q5U Hot Start High‐Fidelity DNA polymerase with Q5U reaction buffer (New England Biolabs, Ipswich, MA, USA; item No. M0515), 200 µM dNTPs, 0.5 µM of each primer (Integrated DNA Technologies, Coralville, IA, USA). To carry out the reaction, an Applied Biosystems ProFlex thermal cycler (Thermo Fisher Scientific, Wilmington, DE, USA) was used with the following conditions: initial denaturation at 98°C for 30 s, 35 cycles consisting of denaturation at 98°C for 10 s, annealing at 64°C for 20 s, and extension at 68°C for 60 s, then final extension at 68°C for 5 min and hold at 4°C. Agarose gel electrophoresis was used to confirm the presence of amplified products and the concentration was assessed by spectrophotometry. Sample purification was performed using the NucleoMag kit (Macherey‐Nagel, Düren, Germany; Item no. 744970) with a 0.8 bead to sample ratio. Sequencing was performed on an Applied Biosystems 3730 DNA Analyzer using the BigDye Terminator V3.1 Cycle Sequencing Kit in the Genomics Core at the University of Guelph. The percentage of methylation of the eight CpG sites was determined using the BiQ Analyzer (Max Planck Institute for Informatics, Saarbrüken, Germany) [30].

### 2.6 Statistical Analyses

SAS Version 9.4 software (SAS Institute Inc., Carey, NC, USA) was used for all ANOVA and correlation analyses. Model assumptions for one‐way ANOVA were assessed using the Shapiro–Wilk test for normality and Levene's test for homogeneity of variance.

For our primary outcome measure, insulin resistance by proxy of HOMA‐IR, a minimum sample size of 10 mice per group was deemed sufficient to detect a 10% difference between groups at a significance level of *p* < 0.05 and a power of 0.80 (by G Power software, version 3.1) [12, 31]. Secondary measures were the expression and methylation of genes in the insulin signaling pathway.

All measures were analyzed by one‐way ANOVA followed by Tukey‐Kramer's post hoc, adjusted for multiple comparisons, for significant effect. Correlations for DNA methylation with mRNA levels, body weight, adiposity fasting insulin, and HOMA‐IR were performed using Pearson's correlation coefficient. Outlier identification and exclusion was performed by robust regression analysis. Statistical significance was defined as *p* < 0.05.

## 3 Results

### 3.1 Body Weight and Adiposity

**FIGURE 2:**
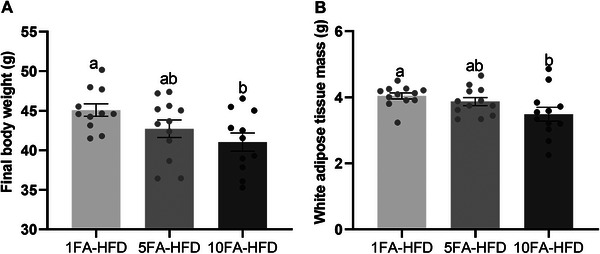
Body weight and adiposity. (A) Final body weight and (B) white adipose tissue mass. *n* = 10–12/group. A one‐way ANOVA was conducted, followed by Tukey‐Kramer post hoc for significant effects. Data presented as means ± S.E.M. ^a,b^Significantly different at *p* < 0.05.

At sacrifice, 10FA‐HFD mice had 9% lower body weight (*p* = 0.028; Figure 2A). While body weight of 5FA‐HFD mice was also reduced, it did not differ from those in 1FA‐HFD or 10FA‐HFD groups (*p* > 0.05). Total white adipose tissue mass (total adiposity), the sum of epididymal and inguinal fat pads, was 13% lower in 10FA‐HFD mice (*p* = 0.033; Figure 2B). 5FA‐HFD mice also had lower total adiposity but did not differ from 1FA‐HFD or 10FA‐HFD mice (*p* > 0.05). Food intake and caloric intake did not differ between groups (*p* > 0.05; Figure S1).

### 3.2 Insulin Resistance

**FIGURE 3:**
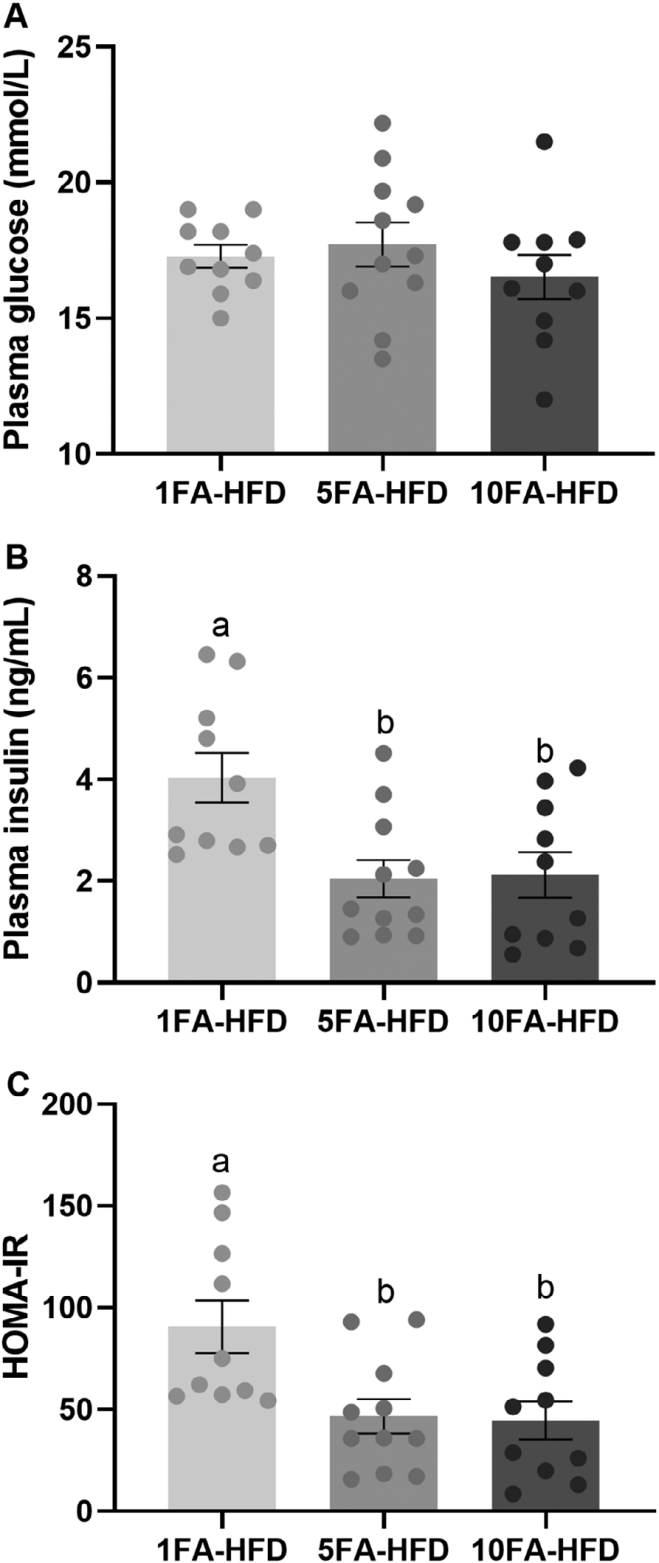
Fasting plasma glucose and insulin concentrations and HOMA‐IR. (A) Fasting plasma glucose, (B) fasting plasma insulin, (C) HOMA‐IR. *n* = 10–11/group. A one‐way ANOVA was conducted followed by Tukey‐Kramer post hoc for significant effects. Data presented as means ± S.E.M. ^a,b^Significantly different at *p* < 0.05.

After 15 weeks on the diets, fasting plasma glucose concentrations did not differ between groups (*p* > 0.05; Figure 3A). However, 5FA‐HFD mice had 49% lower fasting plasma insulin concentrations (*p* = 0.009; Figure 3B), and a 49% lower HOMA‐IR compared to the control (*p* = 0.014; Figure 3C). Similarly, the 10FA‐HFD mice had 47% lower fasting plasma insulin (*p* = 0.014; Figure 3B) and 51% lower HOMA‐IR (*p* = 0.012; Figure 3C).

**FIGURE 4:**
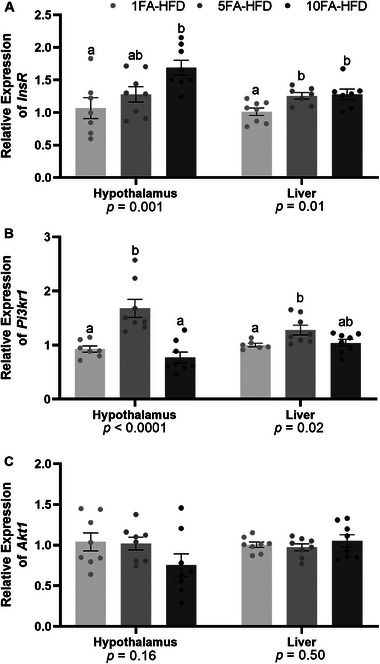
Expression of *InsR*, *Pi3kr1*, and *Akt1* in the hypothalamus and liver. (A) *InsR*, (B) *Pi3kr1*, (C) *Akt1*. *n* = 6–8/group. A one‐way ANOVA was conducted (*p* value presented below each gene target) followed by Tukey‐Kramer post hoc for significant effects. Data presented as means ± S.E.M. ^a,b^Significantly different at *p* < 0.05.

Relative mRNA expression levels of insulin receptor (*InsR*) gene were higher in the hypothalamus of 10FA‐HFD mice (58%, *p* = 0.008; Figure 4A), and in the liver of 5FA‐HFD (24%, *p* = 0.035) and 10FA‐HFD (26%, *p* = 0.021) mice (Figure 4A). In the hypothalamus of 5FA‐HFD, mRNA expression of *InsR* was intermediate but not different from either 1FA‐HFD or 10FA‐HFD (*p* > 0.05, Figure 4A).

In both the hypothalamus and liver, phosphatidylinositol 3‐kinase regulatory subunit 1 (*Pik3r1*) gene expression was upregulated in 5FA‐HFD mice compared to the control (75%, *p* < 0.0001 and 28%, *p* = 0.02, respectively, Figure 4B). Downstream AKT serine/threonine kinase 1 (*Akt1*) mRNA expression was not different between groups in either hypothalamus or liver (*p* > 0.05, Figure 4B).

### 3.3 Hepatic One‐Carbon Products

Concentrations of 1C products were measured in the liver (Table 1). *S*‐adenosylmethionine was significantly higher in the 10FA‐HFD (19%, *p* = 0.013), while 5FA‐HFD mice did not differ from control (*p* > 0.05) but were 19% lower than 10FA‐HFD groups (*p* = 0.014). Concentrations of SAH were significantly higher in the 5FA‐HFD group (27%, *p* = 0.038), while SAH in the 10FA‐HFD mice was not different from either 1FA‐HFD nor 5FA‐HFD (*p* > 0.05). Cystathionine was higher in the 5FA‐HFD group (42%, *p* = 0.049), while concentrations in 10FA‐HFD mice did not differ from 1FA‐HFD nor 5FA‐HFD mice (*p* > 0.05). Hepatic methionine concentrations did not differ between groups (*p* > 0.05).

**TABLE 1:** Hepatic one‐carbon metabolites.

Metabolites (nmol/g tissue)	1FA‐HFD	5FA‐HFD	10FA‐HFD	*p* value
Methionine	53.02 ± 4.39	60.86 ±3.26	52.15 ± 4.00	0.264
S‐adenosylmethionine	59.88 ± 2.76^a^	59.85 ± 2.30^a^	71.32 ± 2.51^b^	**0.006**
S‐adenosylhomocysteine	24.90 ± 0.81^a^	34.25 ± 3.46^b^	30.39 ± 1.30^ab^	**0.031**
Cystathionine	6.81 ± 0.59^a^	9.65 ± 1.05^b^	6.84 ± 0.52^ab^	**0.028**

*Note*: *n* = 11–12/group. Data was analyzed by one‐way ANOVA. Significant effects were analyzed by Tukey‐Kramer post‐hoc test. Data presented as means ± S.E.M.

^a,b^Significantly different at *p* < 0.05.

**FIGURE 5:**
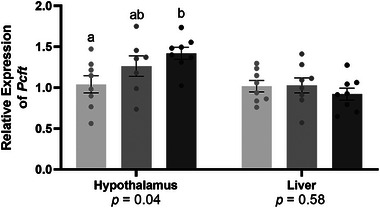
Expression of proton coupled folate transporter (*Pcft*) in the hypothalamus and liver. *n* = 6–8/group. A one‐way ANOVA was conducted (*p* value presented below each gene target) followed by Tukey‐Kramer post hoc, adjusted for multiple comparisons, for significant effects. Data presented as means ± S.E.M. ^a,b^Significantly different at *p* < 0.05.

To assess the potential role of FA in regulating these changes, mRNA levels of proton‐coupled folate transporter (*Pcft*), a folate transporter with high affinity for FA and reduced folate forms [32], were measured. In the hypothalamus, *Pcft* was significantly upregulated in the 10FA‐HFD mice (37%, *p* = 0.034; Figure 5). Whereas expression of *Pcft* in 5FA‐HFD mice did not differ significantly from 1FA‐HFD or 10FA‐HFD (*p* > 0.05). In the liver, *Pcft* was not affected by FA (*p* > 0.05, Figure 5).

### 3.4 DNA Methylation of Insulin Receptor Gene

To assess whether gene expression may be regulated by changes in DNA methylation, we targeted *InsR* for gene‐specific bisufite sequencing as it was most responsive to 5‐ and 10‐folds FA supplementation. Details on the forward and reverse primers, designed in MethPrimer (https://www.urogene.org/methprimer/) [33] and the targeted region of the genome are provided in Table S3.

**FIGURE 6:**
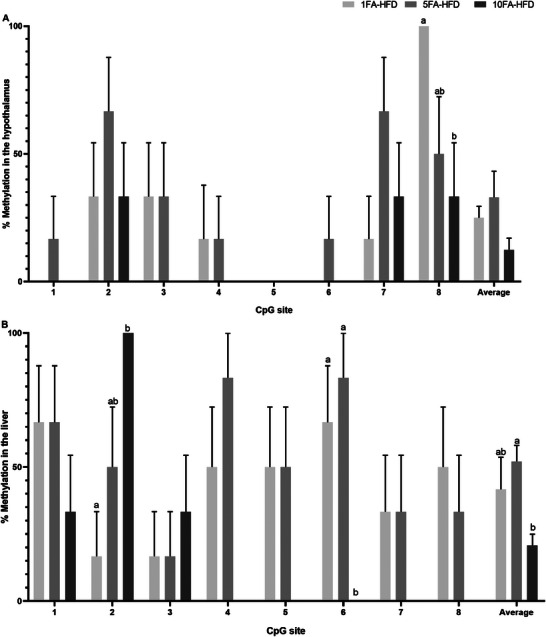
Bisulfite sequencing of *InsR* gene in the promoter region of individual CpG sites and their average in the (A) hypothalamus and (B) liver. *n* = 6/group. A one‐way ANOVA was conducted, followed by Tukey‐Kramer post hoc, adjusted for multiple comparisons, for significant effects. Data presented as means ± S.E.M. ^a,b^Significantly different at *p* < 0.05.

In the hypothalamus, methylation was assessed across eight CpG sites, wherein only one was affected. This CpG site had a step‐down effect in percent methylation with 10FA‐HFD mice having significantly lower methylation compared to the 1FA‐HFD mice (67%, *p *= 0.045, Figure 6A). Average methylation in the hypothalamus showed similar trends to that observed in the liver, with 5‐fold FA mice having the greatest percent methylation and 10‐fold FA mice having the lowest, although there were no statistical differences between groups (*p* > 0.05).

In the liver, within the same region of *InsR*, 2 CpG sites were affected (Figure 6; *p* < 0.05). 10FA‐HFD mice had ∼500% higher methylation at CpG site 2 (*p* = 0.006) and 67% lower methylation at CpG site 6 (*p* = 0.021), compared to the control. 10FA‐HFD mice also had 100% lower methylation at CpG site 6 (*p* = 0.005) when compared to the 5FA‐HFD group. On average, although percent methylation did not differ across groups compared to the control (*p* > 0.05), 5FA‐HFD mice trended toward higher methylation while 10FA‐HFD mice trended toward lower percent methylation. When averaged across the eight CpG sites, 5FA‐HFD mice had 150% higher methylation compared to 10FA‐HFD mice (*p* = 0.004).

In hepatic tissue, DNA methylation of CpG 2 was positively correlated with mRNA levels (*r* = 0.66, *p* = 0.02, Table 2), as well as negatively correlated with body weight (*r* = –0.71, *p* = 0.01) and adiposity (*r* = –0.62, *p* = 0.03). CpG 2 methylation was not correlated to fasting insulin concentrations or HOMA‐IR (*p* > 0.05). Methylation at CpG 6 in the liver was positively correlated with adiposity (*r* = 0.68, *p* = 0.0002, Table 2). In the hypothalamus, CpG 8 was positively correlated with adiposity (*r* = 0.58, *p* = 0.049, Table 2), and in the liver this site was positively correlated with insulin (*r* = 0.72, *p* = 0.009), HOMA‐IR (*r* = 0.75, *p* = 0.005), and adiposity (*r* = 0.71, *p* = 0.01). The average of CpG sites did not correlate with mRNA levels or metabolic phenotype (*p* > 0.05, Table 2).

**TABLE 2:** Associations between *InsR* methylation, expression, and metabolic phenotype.

	Hypothalamus		Liver	
Correlations	*r*	*p* value	*r*	*p* value
CpG 2 %methylation				
* InsR* mRNA level	−0.07	0.82	0.66	**0.02**
Fasting insulin (mmol/L)	−0.09	0.77	−0.24	0.45
HOMA‐IR	−0.06	0.84	−0.21	0.52
FBW (g)	0.11	0.73	−0.71	**0.01**
Total adiposity (g)	0.39	0.20	−0.62	**0.03**
CpG 6 %methylation				
* InsR* mRNA level	−0.25	0.32	−0.20	0.42
Fasting insulin (mmol/L)	−0.28	0.26	0.39	0.11
HOMA‐IR	−0.27	0.27	0.41	0.09
FBW (g)	0.08	0.75	0.11	0.68
Total adiposity (g)	0.22	0.38	0.68	**0.002**
CpG 8 %methylation				
* InsR* mRNA level	−0.34	0.28	−0.43	0.19
Fasting insulin (mmol/L)	0.18	0.57	0.72	**0.009**
HOMA‐IR	0.22	0.57	0.75	**0.005**
FBW (g)	0.21	0.52	0.36	0.24
Total adiposity (g)	0.58	**0.049**	0.71	**0.01**
Average CpG %methylation				
* InsR* mRNA level	−0.50	0.10	0.07	0.82
Fasting insulin (mmol/L)	−0.49	0.11	0.13	0.69
HOMA‐IR	−0.44	0.16	0.15	0.64
FBW (g)	−0.07	0.83	0.42	0.17
Total adiposity (g)	0.33	0.30	0.54	0.07

*Note*: Data from significantly different groups were pooled for correlation analysis. Associations were analyzed by Pearson's correlation test, with the Pearson correlation coefficient depicted for each correlation.

### 3.5 Expression of DNA Methyltransferase Genes

**FIGURE 7:**
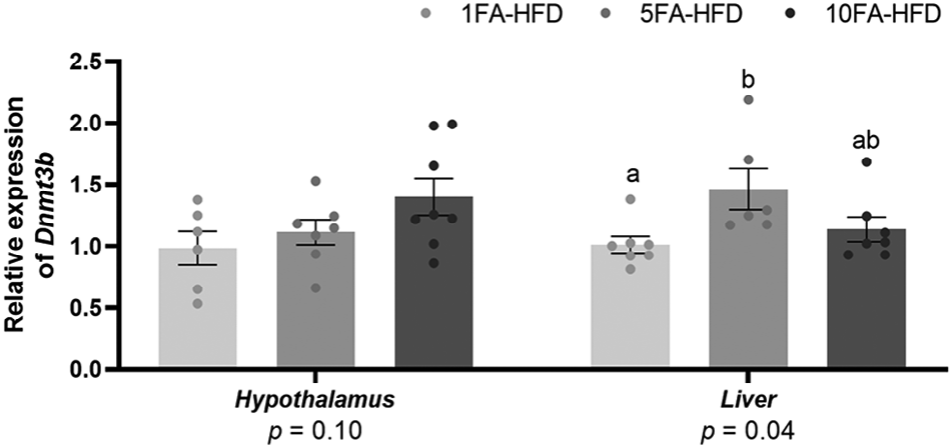
Expression of DNA methyltransferase 3b (*Dnmt3b*) in the hypothalamus and liver. *n* = 6–8/group. A one‐way ANOVA was conducted (*p* value presented below each gene target) followed by Tukey‐Kramer post‐hoc, adjusted for multiple comparisons, for significant effects. Data presented as means ± S.E.M. ^a,b^Significantly different at *p* < 0.05.

To assess if FA impacted the expression of the enzymes mediating DNA methylation reactions, relative mRNA expression levels of hypothalamic and hepatic DNA methyltransferases (*Dnmts*) were measured. Maintenance *Dnmt1* gene expression was not affected in the hypothalamus or liver (*p* > 0.05; Figure S2). Similarly, de novo *Dnmt3a* expression was not affected by FA in the hypothalamus and liver (*p* > 0.05; Figure S2). However, de novo *Dnmt3b* expression was higher in the liver of 5‐fold FA‐fed mice compared to the control (45%, *p* = 0.032; Figure 7). Expression of *Dnmt3b* in 10‐fold mice was intermediate to control and 5FA‐HFD mice (*p* > 0.05). In the hypothalamus, *Dnmt3b* was not affected in either 5‐ or 10‐fold FA groups (*p* > 0.05; Figure 7).

## 4 Discussion

The results of this study support our hypothesis that modifying 1C metabolism through FA supplementation reduces IR in DIO mice by differentially altering methylation patterns of IR‐related genes in the hypothalamus and liver. By supplementing mice with FA, we have several lines of evidence showing that metabolic dysregulation in DIO mice can be reduced through its impact on 1C metabolism and gene expression and function in the hypothalamus and liver. To our knowledge, this is the first report exploring how FA at differing doses differentially induces tissue‐specific expression and methylation of IR‐related genes postweaning.

We observed that 10‐fold FA supplemented mice had lower body weight, consistent with some reports [34, 35], although others have observed no change [13, 21, 22], or increased body weight [36]. These inconsistencies may be attributable to differences in the rodent model [36], dietary fat content [21, 22, 36], and intervention duration [21]. Moreover, 10‐fold FA reduced total white adipose tissue mass, as others have reported previously [12, 13]. When total adipose tissue mass was adjusted to final body weight, this effect was abolished, suggesting the lower body weight is mediated by reductions in adiposity. These reductions in body weight and adiposity were not attributed to lower food intake in FA supplemented groups. Both 5‐ and 10‐fold FA supplemented mice had ∼50% lower HOMA‐IR scores, compared to the control. Interestingly, 5‐fold mice showed similar reductions in insulin and HOMA‐IR despite not having as strong of an effect on body weight and adiposity, suggesting that other factors are contributing to this metabolic improvement. Others have reported increasing the FA content (2.5–20‐fold AIN‐93) in the diets of adult DIO rodents lowered IR [12, 13], and glycemic control [12]. A study in HFD‐fed male mice found that supplementing FA to the drinking water altered DNA methylation patterns in epididymal fat pads, where many of the affected genes were associated with IR [12]. Similarly, a recent meta‐analysis of human studies showed FA supplementation (>5 mg, >12.5‐fold RDA) was associated with reduced fasting blood glucose, fasting insulin, and homeostatic model of assessment for IR (HOMA‐IR) [37]. However, the underlying mechanisms are uncertain.

The current study shows that these metabolic effects may be mediated through 1C metabolism and the resulting changes in methylation of regulatory genes in the hypothalamus and liver. In the hypothalamus, we observed a step‐down effect in the methylation levels of CpG site 8, with a stepwise increase in the mRNA expression levels in 5‐ and 10‐fold FA supplemented mice. The lower amount of methylation in the *InsR* is consistent with our previous study that found that adding 10‐fold methyl vitamins (B6, B12, and FA) to the gestational diet led to lower average methylation in hypothalamic tissue of the male offspring [11]. Neonatal overfed and adult offspring rats fed high‐fat gestational diets had higher promoter methylation of hypothalamic *InsR* [17, 18]. Thus, lower methylation in the promoter region of hypothalamic *InsR* demonstrates potential reversal from the effects of HFD‐feeding [17]. However, methylation at CpG site 8 of *InsR* did not correlate with its mRNA expression which may suggest that the epigenetic regulation of this gene by DNA methylation is outside of the portion of the promoter region targeted in this study.

In the liver, both average and specific CpG sites were affected, with both increased and decreased methylation in response to FA supplementation. Both hyper‐ and hypo‐methylation of CpG sites within promoter regions can occur and still be functionally relevant due to their impact on gene expression, even if they appear contradictory. In some cases, hypermethylation paradoxically activates genes; the exact mechanisms are still being explored but may involve alterations in chromatin structure or recruitment of transcription factors [38, 39]. Nevertheless, the significant relationship between CpG‐site specific methylation changes and metabolic phenotype revealed by correlation analyses is a noteworthy finding. For instance, there was a positive association observed between methylation at CpG 2 and hepatic mRNA levels of *InsR*. Methylation at CpG 2 in the liver was also negatively correlated with body weight and adiposity, whereas methylation at CpG 8 in the liver was negatively associated with fasting insulin concentrations and HOMA‐IR suggesting that methylation of this gene may be contributing to changes in metabolic parameters and phenotype. In line with this, methyl donor (betaine, choline, FA, and B12) supplementation to high‐fat‐high‐sucrose diets in adult male Wistar rats both increased and decreased site‐specific hepatic methylation of several lipid metabolism genes was correlated with various anthropometrics [40]. This highlights the complexities of methylation patterns and their impact on gene expression and metabolic function.

The effects of FA on gene methylation were both site‐ and tissue‐specific. Overall, there were fewer affected CpG sites in the hypothalamus compared to the liver, although differences in *InsR* mRNA expression levels between 10‐fold FA and control were more pronounced in the hypothalamus. Fewer changes in methylation patterns in the hypothalamus may also explain the lack of changes in *Dnmt* levels in the tissue between treatment groups. Despite this, CpG sites were affected in both tissues demonstrating that modification of the 1C metabolism through FA altered the epigenome across both the hypothalamic and hepatic tissues associated with the observed phenotypic changes.

Interestingly, there were discrepancies with some markers not showing a dose response trend, particularly with the effects of folic acid on SAM/SAH, *Dnmt* expression, and CpG methylation. The relationship between high folic acid intake DNMT enzymes and SAM/SAH levels is complex and multifaceted. Some research suggests that high folic acid intake can stimulate the expression and activity of DNMT enzymes, potentially leading to increased DNA methylation [41]. Other studies found that folic acid supplementation resulted in lower mRNA expression levels of *Dnmt1* and *Dnmt3a* in the brain of mice [42] and DNMT3a protein expression in colon cancer cell lines [43]. It is plausible that the effect of folic acid on DNMT enzymes may depend on the specific DNMT isoform (e.g., DNMT1, DNMT3A, and DNMT3B), the cell/tissue type, and the overall folate status. This may suggest that although 10‐fold FA had the strongest and most comprehensive effects, it possibly reached a point of saturation of DNMTs with potentially less utilization of methyl groups from SAM, as reflected in the nonsignificant difference in hepatic SAH concentrations and lower CpG methylation in 10‐fold FA supplemented mice compared to the control. In contrast, 5‐fold FA group had higher SAH and higher average CpG methylation of the *InsR* promoter region with upregulation of *Dnmt3b*. This observation is supported by others that have reported FA supplementation to increase the expression and activity of DNMTs [41]. It is also possible that there was a shift toward other methylation reactions, such as RNA methylation, which has previously been reported to be affected by folic acid supplementation [44].

Some limitations were present in this study. We did not tease apart the effects of FA on metabolism from those driven by the lower body weight. Others, however, have reported improvements in metabolic phenotype, despite no changes in body weight and adiposity [13, 21, 22]. Thus, it is plausible that the effects are not regulated entirely by changes in body weight and fat accumulation but may also be mediated by the effects of FA on metabolism directly. Given the limited amount of hypothalamic tissue available for analysis, 1C metabolites were only measured in hepatic tissues. We also did not measure folate concentrations in blood and tissues as the limited sample amount did not allow for their accurate measurement. While several CpG sites were correlated with *InsR* gene expression and phenotypic measures, it is worth noting that the eight CpG sites captured only quantify a small portion of the *InsR* gene and epigenetic regulation could also be occurring within regions not measured in this study. Furthermore, while changes in methylation of the *InsR* gene were associated with reduced IR and body weight, this current study cannot address if these changes are directly caused by gene methylation. Future work should examine the direct mechanisms through inhibition of DNMTs on metabolic phenotype and gene expression.

This study adds support to using FA during the postweaning period to improve metabolic parameters in obesity. As gene‐specific DNA methylation was associated with improved metabolic phenotype, it highlights the potential for utilizing dietary strategies to modify the epigenome to reduce or prevent obesity development. Gaining a greater understanding of the underlying mechanism(s) driving these metabolic improvements will lend support for clinical translatability.

## Supporting information




**Supporting File 1**: mnfr70181‐sup‐0001‐SuppMat.docx.

## Data Availability

The data that support the findings of this study are available from the corresponding author upon reasonable request.
