# Supplementary material for: Folic Acid Reduces Insulin Resistance in Mice With Diet‐Induced Obesity by Altering One‐Carbon Metabolism and DNA Methylation Patterns of Hypothalamic and Hepatic Insulin Receptor Gene
Source: Mol Nutr Food Res. 2025 Jul 16;69(20):e70181. doi: 10.1002/mnfr.70181 (PMC12538536; doi:10.1002/mnfr.70181)
Supplement: Supplementary file 1 — Supporting File 1: mnfr70181‐sup‐0001‐SuppMat.docx. [file MNFR-69-e70181-s001.docx]

Supporting Information:

**Supplemental Table 1. Diet Composition.**

|  | **1FA-HFD** | **5FA-HFD** | **10FA-HFD** |
| --- | --- | --- | --- |
|  | g/kg (kcal%) | | |
| Protein | 203 (20) | 203 (20) | 203 (20) |
| Carbohydrate | 356.3 (35) | 356.3 (35) | 356.3 (35) |
| Fat | 202.5 (45) | 202.5 (45) | 202.5 (45) |
| Ingredients | g | | |
| Casein | 200 | 200 | 200 |
| L-cystine | 3 | 3 | 3 |
|  |  |  |  |
| Corn Starch | 72.8 | 72.8 | 72.8 |
| Maltodextrin 10 | 100 | 100 | 100 |
| Sucrose | 172.8 | 172.8 | 172.8 |
|  |  |  |  |
| Cellulose, BW200 | 50 | 50 | 50 |
|  |  |  |  |
| Lard | 177.5 | 177.5 | 177.5 |
| Soybean Oil | 25 | 25 | 25 |
|  |  |  |  |
| t-Butylhydroquinone | 0.014 | 0.014 | 0.014 |
|  |  |  |  |
| Mineral Mix (S10022C, 10X) | 35 | 35 | 35 |
| Vitamin Mix (V10037C, 10X) | 1 | 1 | 1 |
| **Folic Acid, added** | **0** | **0.008** | **0.018** |
|  |  |  |  |
| Choline Bitartrate | 2.5 | 2.5 | 2.5 |
| **Total** | 839.614 | 839.622 | 839.632 |

**Supplemental Table 2. TaqMan® Gene Expression Assays.**

| Pathway/Gene | Assay ID |
| --- | --- |
| **PI3K/AKT Signalling** | |
| Insulin receptor, *Insr* | Mm01211875_m1 |
| Phosphoinositide-3-kinase regulatory subunit 1, *Pik3r1* | Mm01282781_m1 |
| AKT serine/threonine kinase 1, *Akt1* | Mm01331626_m1 |
| **DNA methyltransferases** | |
| DNA methyltransferase 1, *Dnmt1* | Mm01151063_m1 |
| DNA methyltransferase 3a, *Dnmt3a* | Mm00432881_m1 |
| DNA methyltransferase 3b, *Dnmt3b* | Mm01240113_m1 |
| **One-Carbon Metabolism and Folate Uptake** | |
| Choline dehydrogenase, *Chdh* | Mm00549261_m1 |
| Glycine N-methyltransferase, *Gnmt* | Mm00494688_m1 |
| Proton-coupled folate transporter, *Pcft* (i.e., *Slc46a1*) | Mm00546630_m1 |
| **Endogenous Controls** | |
| TATA box binding protein, *Tbp* | Mm00446973_m1 |
| Beta-2-microglobulin, *B2m* | Mm00437762_m1 |

**Supplemental Table 3. Insulin receptor primer for methylation analysis.**

|  |  |
| --- | --- |
| **Forward Primer** | 5' TTATAGTAGTAGGAGTGTTGGGATT 3' |
| **Reverse Primer** | 5' CACCACCAACCCAAATAC 3' |
| **Target Region*** | CAGGTCACCCAGGCC**CG**GTCC**CG**TGCC**CG**CAATCCCAGAGTCCAGAGCTCAC**CGCG**GTG**CG**TCTCCTGTC**CG**GGTCTCCTGCTTCTGCCCTC**CG**TGCAGCTCT |

*Bolded text indicates targeted CpG sites for methylation analyses.

| **A**  | **B**  |
| --- | --- |

**Supplemental Figure 1. Cumulative food and caloric intake**. Food intake (A) and caloric intake (B) are represented as per cage intake measured once weekly over the duration of the study.

| **A**  **** |
| --- |
| **B**  **** |

**Supplemental Figure 2. Expression of DNA methyltransferase 1 and 3a in hypothalamus and liver. A) *Dnmt1*, n = 6-8/group, B) *Dnmt3a*, n = 6-8/group.** A one-way ANOVA was conducted (*p*-value presented below each gene target) followed by Tukey-Kramer post-hoc, adjusted for multiple comparisons, for significant effects. ^a,b^Significantly different at *p* < 0.05.
